# Detection of SARS-CoV-2 in high-efficiency particulate air (HEPA) filters of low-cost air purifiers in community-based organizations

**DOI:** 10.1007/s10661-023-11950-y

**Published:** 2023-10-14

**Authors:** Rachel D. Clarke, Nana Aisha Garba, Manuel A. Barbieri, Leonardo Acuna, Marianna Baum, Maribel Saad Rodriguez, Hansel Frias, Paulina Saldarriaga, Troy Stefano, Kalai Mathee, Giri Narasimhan, David R. Brown

**Affiliations:** 1 Department of Humanities Health, and Society, Herbert Wertheim College of Medicine, Florida International University, Miami, FL USA; 2 Department of Biological Sciences, Florida International University, Miami, FL USA; 3 Biochemistry PhD Program, Florida International University, Miami, FL USA; 4 Department of Dietetics and Nutrition, Robert Stempel College of Public Health and Social Work, Florida International University, Miami, FL USA; 5 Department of Environmental Health Sciences, Robert Stempel College of Public Health and Social Work, Florida International University, Miami, FL USA; 6 Department of Human and Molecular Genetics, Herbert Wertheim College of Medicine, Florida International University, Miami, FL USA; 7 Biomolecular Sciences Institute, Florida International University, Miami, FL USA; 8 Bioinformatics Research Group (BioRG), Knight Foundation School of Computing and Information Sciences, Miami, FL USA

**Keywords:** COVID-19, SARS-CoV-2, Environmental monitoring, Indoor air, HEPA filters

## Abstract

This study aims to investigate the presence of SARS-CoV-2 in public spaces and assess the utility of inexpensive air purifiers equipped with high-efficiency particulate air (HEPA) filters for viral detection. Samples were collected from six community-based organizations in underserved minority neighborhoods in Northwest Miami, Florida, from February to May 2022. Reverse transcription–quantitative polymerase chain reaction (RT-qPCR) was used to detect SARS-CoV-2 in air purifier filters and surface swabs. Among 32 filters tested, three yielded positive results, while no positive surface swabs were found. Notably, positive samples were obtained exclusively from child daycare centers. These findings highlight the potential for airborne transmission of SARS-CoV-2 in indoor air, particularly in child daycare centers. Moreover, the study demonstrates the effectiveness of readily available HEPA filters in detecting the virus. Improving indoor ventilation and implementing air filtration systems are crucial in reducing COVID-19 transmission where people gather. Air filtration systems incorporating HEPA filters offer a valuable approach to virus detection and reducing transmission risks. Future research should explore the applicability of this technology for early identification and mitigation of viral outbreaks.

## Introduction

The COVID-19 pandemic caused by SARS-CoV-2 presented significant challenges for public health, particularly indoors where transmission occurs through respiratory fluids and airborne routes (Centers for Disease Control and Prevention, n.d.; Prather et al., 2020; Tabari et al., 2020). To mitigate these risks, supplemental measures such as the increased natural ventilation and the use of portable air cleaners (PACs) with high-efficiency particulate air (HEPA) filters and heating, ventilation, and air conditioning (HVAC) system upgrades have been proposed (Lindsley et al., 2021). Early in the pandemic, a number of studies demonstrated the presence of SARS-CoV-2 in air samples, using specialized air sampling equipment in hospital settings (Chia et al., 2020; Faridi et al., 2020; Liu et al., 2020). This study aimed to assess the effectiveness of readily available low-cost air purifiers with HEPA filters in detecting SARS-CoV-2 in community-based settings, with a particular emphasis on early identification and containment of potential outbreaks. The presence of the virus was investigated on high contact surfaces such as door handles, toilet handles, elevator call buttons, sink fixtures, and children’s tables and within air purifier filters at various community-based organizations in underserved minority neighborhoods in North Miami-Dade County, Florida. Through the development and testing of a technique to detect SARS-CoV-2 RNA in HEPA filters under realistic conditions in a diverse range of community settings, this study aimed to provide insights into the efficacy of small personal air purifiers (priced at $35 USD) and replacement filters ($16 USD) as a cost-effective alternative to expensive air sampling techniques used in previous research (Chia et al., 2020; Faridi et al., 2020; Liu et al., 2020), thereby facilitating widespread accessible monitoring strategies for respiratory viruses.

## Materials and methods

### Partner engagement

This study leverages community infrastructure, outreach staff, and trust-based relationships of the Green Family Foundation Neighborhood Health Education Learning Program (NeighborhoodHELP), an academic–community partnership addressing the social determinants of health (SDOH) at the Florida International University Herbert Wertheim College of Medicine (Greer et al., 2018). The NeighborhoodHELP outreach team worked with representatives of partner organizations to identify interested participants and gather information about their facility layout, utilization, and areas of congregation.

### Description of collection sites

Six partner sites, including a community health clinic, a faith-based organization, a public library, and three child daycare/educational facilities, were included. High-traffic areas such as bathrooms, waiting rooms, break/lunchrooms, classrooms, group meeting rooms, and offices were selected based on discussions with partner representatives. The facilities accommodated between 8 and 1220 daily visitors, with high-traffic days typically occurring Monday through Friday.

### Sample collection

**Fig. 1:**
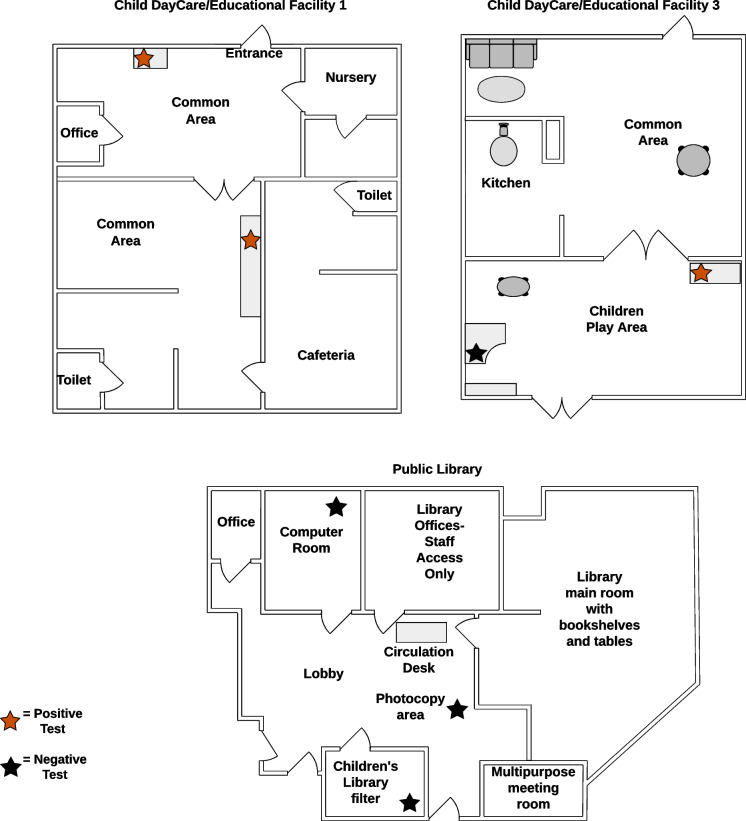
Floor plans of sites with positive tests and one with all negative tests

During the initial visit to each site, discussions with the site representatives guided the selection of surfaces and areas for sample collection. Samples were collected between February 3rd, 2022, and May 5th, 2022, and were dropped off at the lab within 7 days after collection. Surface samples were collected from specific surfaces using the “BD Universal Viral Transport Kit with a flexible minitip flocked swab” (BD Universal Viral Transport System - 220531 | BD, n.d.). Surfaces were vigorously swabbed with a sterile collection swab that was dipped into the viral transport medium in the kit and placed into a collection tube after swabbing. Air sampling involved placing ProBreeze PB-P02 Mini Air purifiers with multi-layer filters in high-traffic areas to capture potential viral aerosols (Fig. 1). Based on the manual for the ProBreeze PB-P02 Mini (PB-P02-US - 3-in-1 Mini Air Purifier – Pro Breeze Help Centre, n.d.), it is estimated that the air moved through the filter at 7CFM.

HEPA filters were removed during subsequent visits (between 2 and 4 weeks), placed in a TapeSeal 95kPA specimen transport bag, sealed, and transported to the laboratory. In some locations, multiple rounds of sampling were performed, with purifiers wiped with disinfectant and replacement filters placed for subsequent collections.

### Laboratory processing

A 15-cm × 2-cm-long strip from the middle of the HEPA filter and a 3-cm × 3-cm square of the pre-filter were cut into small pieces and placed into a 50-mL conical tube with 3 mL of Integrated DNA Technologies Tris EDTA buffer (IDTE) (Buffers and Solutions, n.d.) in a rocking shaker overnight. The mixture was then placed in a syringe filter (13mm in diameter with 0.45μm pores), the plunger was reinserted, and the filter material was compressed to provide the maximum amount of eluent for analysis. Validation for the assay was performed with SARS-CoV-2 virus pipetted on filter material. RNA extraction was conducted using the MagMax^TM^ Viral/Pathogen Nucleic Acid Isolation Kit, and eluted total RNA was used for RT-qPCR for SARS-CoV-2 detection. The RT-qPCR assay used in this study is a laboratory-developed test (LDT) based on the CDC’s 2019-Novel Coronavirus (2019-nCoV) Real-Time RT-PCR Diagnostic Panel (CDC 2019-Novel Coronavirus (2019-NCoV) Real-Time RT-PCR Diagnostic Panel for Emergency Use Only Instructions for Use, n.d.) in a CLIA-certified laboratory. The assay utilized primer and probe sequences identical to those in the 2019-nCov CDC EUA Kit (2019-Novel Coronavirus (2019-NCoV) Real-Time RRT-PCR Panel Primers and Probes, 2020). RT-qPCR was carried out at 25 °C for 2 min, then at 55 °C for 15 min for reverse transcription, followed by 95 °C for 2 min, and then 40 cycles of 95 °C for 3 s, and 55 °C for 30 s using the Taqpath 1-step RT-qPCR master mix. These methods allowed for collection of both surface and air samples and the subsequent detection of SARS-CoV-2 via RT-qPCR.

## Results and discussion

A total of 32 air filter samples were collected from high-traffic areas at the six partner sites and three samples, all from child daycare centers, tested positive for SARS-CoV-2 via RT-qPCR with Cq values ranging of 36.26 to 39.17 for N1 and from 38.03 to 39.55 for N2 (Table 1). Two positive samples were collected at a child daycare/educational facility in an activity room, and a third positive sample was collected at a different child daycare facility (Figure 1, Table 2). Of the 35 surface swabs collected, none tested positive for SARS-CoV-2.

**Table 1:** Cq values for control vs. positive test samples

Sample #	1		2		3	
Viral region	N1	N2	N1	N2	N1	N2
Positive control	27.94	28.28	25.19	25.15	25.19	25.15
Positive extraction control Cq	31.91	33.18	29.58	30.34	29.58	30.34
Test sample Cq	39.17		36.26	38.03		39.55
Interpretation	Virus detected		Virus detected		Virus detected	

*N1* SARS-COV2 nucleocapsid region 1

*N2* SARS-COV2 nucleocapsid region 2

**Table 2:** Community facility sample locations and test results

Location type	COVID-19 preventive measures employed	Estimated weekly traffic (people)	Room where sample was collected	Specific location where sample was collected	Type of sample collection	# of negative samples	# of positive samples
Community health clinic (CHC)	• Masks required at all times indoors	225−250	Clinic entry area and bathroom	• Under chair near elevator	Filter	1	0
				• Entrance door interior handle • Entrance door exterior handle • Pharmacy keypad • Bathroom door • Elevator hall call button • Elevator car operating panel	Swab	10	0
			Waiting areas	• Adjacent to reception desks • Below wall-mounted TV	Filter	6	0
Public library (PL)	•Mask required at all times indoors	150−200	Open area	• Adjacent to copier machine	Filter	2	0
				• Various surface	Swab	6	0
Faith-based organization (FBO)	• Teachers required to wear masks	500−750	Children’s play and eating areas	• Adjacent to window	Filter	1	0
				• Main doorknob • Storage doorknob • Children’s table • Children’s toys	Swab	4	0
			Sanctuary and restroom	• Center of floor	Filter	1	0
				• Microphone tip • Restroom handlebar	Swab	2	0
Child daycare/educational facility 1 (CD1)	•Masks required for adults	60	Entrance area	• Right-side of entrance desk	Filter	3	1
			Classroom and hallway	• Cubby stations	Filter	3	1
				• Sink fixtures • Children’s table • Interior door handle	Swab	3	0
Child daycare/educational facility 2 (CD2)	• N95 masks & personal protective equipment (PPE) for staff • Contactless pick-up & drop-off • Staggered scheduling • Social distancing • Daily temperature checks	257	Entry office area and (small) interior clinic area	• Reception desk • Clinic interior	Filter	5	0
				• Entrance door exterior handles • Login computer adjacent to reception table • Copy machine screen	Swab	3	0
			Main hallway and connected areas	• Hallway • Human resource office	Filter	3	0
				• Employee bathroom toilet flush handles • Classroom entrance door handle	Swab	2	0
			Kitchen	• Countertop	Filter	1	0
				• Interior door handle	Swab	1	0
Child daycare/educational facility 3 (CD3)	• Mask and gloves required for adults	50−100	Children play and dining area with interior bathroom	• Rear wall near bookshelf • Near computer desk	Filter	6	1
				• Bathroom exterior handle • Changing table • Toilet handle • Sink fixtures	Swab	4	0

All portable air cleaners with filters were placed in high-traffic areas in each facility

The positive samples obtained in this study underscore the importance of understanding transmission dynamics in high-traffic areas, especially in child daycare centers and other areas of indoor crowding in lower socioeconomic status and minority neighborhoods. Mask mandates varied during the study period, with adults required to wear masks in the child daycare facilities, while children were not mandated to do so. The one child daycare center that never tested positive had undertaken extensive non-pharmaceutical interventions (NPIs) for risk mitigation. The absence of positive surface swabs suggests that surface transmission may not have been a primary mode in these settings, with close contact, droplets, and aerosols likely playing a more significant role. On the other hand, it may be related to the frequent wipe down with sanitizers common during the pandemic.

Air filtration with HEPA filters provides a potential strategy for reducing the risk of airborne transmission of SARS-CoV-2 in community-based organizations since they are low-cost, easily accessible to the community, and easy to use. HEPA filters have high efficiency in capturing particles as small as 0.3 μm (Chia et al., 2020; Ventilation and Coronavirus (COVID-19) | US EPA, n.d.). Although the size of SARS-CoV-2 respiratory particles is estimated to be smaller, several factors contribute to the entrapment of the smaller viral particles in HEPA filters. For example, entrapment could be the result of the particles carrying SARS-CoV-2 usually being larger than 0.3 μm due to the presence of the respiratory fluids that surround it (Bhat et al., 2022). Another reason may be the Brownian motion effect which allows for the entrapment of smaller particles in HEPA filters due to their entanglement with larger particles such as respiratory droplets or other particles in the air (Hao, 2005; Lee, 2020). Additionally, porous surfaces and desiccated virions on these surfaces are known to decrease virion viability compared to hard surfaces (Hosseini et al., 2021). The constant airflow of air purifier filters may capture and aid in the inactivation of viruses (Berry et al., 2022).

The presence of SARS-CoV-2 in HEPA filters of these mini air purifiers highlights the potential risk of airborne transmission in crowded, unmasked indoor spaces. These findings support previous research emphasizing the importance of improving indoor ventilation and air filtration to reduce the spread of COVID-19. Further research is needed to explore the effectiveness of different air purifiers and filtration systems, especially in child daycare centers, for reducing SARS-CoV-2 transmission in indoor environments. Environmental monitoring for respiratory viruses in public settings gains significance through this study.

The presence of SARS-CoV-2 in HEPA filters of PACs shows their effectiveness in detecting the virus within indoor environments. Recently, others have also reported detecting SARS-CoV-2 with an air pump connected to a filtration membrane (López et al., 2021) and in HEPA filters from commercially available and/or low-cost PACs (Fernández de Mera et al., 2022) and HVAC filters (Pan et al., 2022). HVAC filters have previously been proposed as a source for sampling for airborne viruses in public settings (Goyal et al., 2011).

## Conclusion

The findings demonstrate that even low-cost purifiers with relatively low clean air delivery rates (CADR) can play a valuable surveillance role, making widespread monitoring strategies more accessible and feasible. Past research on aerosol sampling for SARS-CoV-2 focused on hospital settings and used laboratory sampling methods not well suited to community settings (Chia et al., 2020; Faridi et al., 2020; Liu et al., 2020; López et al., 2021; Rahmani et al., 2020). Future research could explore the development of portable devices for on-site virus detection or routine testing of HVAC or PAC filters in selected high-traffic facilities. Rapid and efficient detection of viruses in public settings could aid in early identification and mitigation of outbreaks, reducing the impact of infectious diseases on public health. In addition to wastewater (Hamouda et al., 2021), HVAC systems (Goyal et al., 2011; Pan et al., 2022) and PACs (Fernández de Mera et al., 2022) offer another option for environmental monitoring.

In conclusion, our study provided evidence for the effectiveness of widely accessible and affordable HEPA filters in monitoring the presence of SARS-CoV-2 in community-based settings, particularly in child daycare facilities. These results have significant implications, emphasizing the role of environmental monitoring and the potential for widespread strategies using air purification systems for monitoring respiratory viruses in the urban microbiome.

## Data Availability

The datasets generated during and/or analyzed during the current study are available from the corresponding author on reasonable request.
